# *Yersinia pestis* Endowed with Increased Cytotoxicity Is Avirulent in a Bubonic Plague Model and Induces Rapid Protection against Pneumonic Plague

**DOI:** 10.1371/journal.pone.0005938

**Published:** 2009-06-16

**Authors:** Ayelet Zauberman, Avital Tidhar, Yinon Levy, Erez Bar-Haim, Gideon Halperin, Yehuda Flashner, Sara Cohen, Avigdor Shafferman, Emanuelle Mamroud

**Affiliations:** 1 Department of Biochemistry and Molecular Genetics, Israel Institute for Biological Research, Ness-Ziona, Israel; 2 Department of Biotechnology, Israel Institute for Biological Research, Ness-Ziona, Israel; BMSI-A*STAR, Singapore

## Abstract

An important virulence strategy evolved by bacterial pathogens to overcome host defenses is the modulation of host cell death. Previous observations have indicated that *Yersinia pestis*, the causative agent of plague disease, exhibits restricted capacity to induce cell death in macrophages due to ineffective translocation of the type III secretion effector YopJ, as opposed to the readily translocated YopP, the YopJ homologue of the enteropathogen *Yersinia enterocolitica* O∶8. This led us to suggest that reduced cytotoxic potency may allow pathogen propagation within a shielded niche, leading to increased virulence. To test the relationship between cytotoxic potential and virulence, we replaced *Y. pestis* YopJ with YopP. The YopP-expressing *Y. pestis* strain exhibited high cytotoxic activity against macrophages *in vitro*. Following subcutaneous infection, this strain had reduced ability to colonize internal organs, was unable to induce septicemia and exhibited at least a 10^7^-fold reduction in virulence. Yet, upon intravenous or intranasal infection, it was still as virulent as the wild-type strain. The subcutaneous administration of the cytotoxic *Y. pestis* strain appears to activate a rapid and potent systemic, CTL-independent, immunoprotective response, allowing the organism to overcome simultaneous coinfection with 10,000 LD_50_ of virulent *Y. pestis*. Moreover, three days after subcutaneous administration of this strain, animals were also protected against septicemic or primary pneumonic plague. Our findings indicate that an inverse relationship exists between the cytotoxic potential of *Y. pestis* and its virulence following subcutaneous infection. This appears to be associated with the ability of the engineered cytotoxic *Y. pestis* strain to induce very rapid, effective and long-lasting protection against bubonic and pneumonic plague. These observations have novel implications for the development of vaccines/therapies against *Y. pestis* and shed new light on the virulence strategies of *Y. pestis* in nature.

## Introduction

The genus *Yersinia* is comprised of three human pathogens: *Y. pestis*, *Y. pseudotuberculosis*, and *Y. enterocolitica*. *Yersinia pestis* is the causative agent of plague, an acute and often fatal disease [1]. Bubonic plague, which develops following a bite by an infected flea, and pneumonic plague, which ensues from inhaled bacterial aerosol, are two forms of the disease. *Y. enterocolitica* and *Y. pseudotuberculosis* are fecal-oral enteropathogens that cause invasive gastrointestinal diseases that are usually overcome by the host. The three pathogenic *Yersinia* species share a common type III secretion system (TTSS) that is essential for virulence. The TTSS system is encoded by a 70 kb plasmid and its production is induced by temperature elevation to 37°C. It interacts with the eukaryotic host cell [2] to form a translocation apparatus for injecting effector proteins into the cytosol. These effector proteins, known as *Yersinia* outer proteins (Yops), act to down-regulate host defense mechanisms. The major known effects of Yops are counteraction of host innate immune cell function such as pathogen ingestion and destruction within the phagosome, induction of pro-inflammatory cytokines and subsequent stimulation of the adaptive immune system [3]. Numerous studies have shown that the major mechanism of action of Yop effector proteins is the disruption of the target cell signaling network and cytoskeleton rearrangement, which are necessary for phagocytosis by host macrophages and polymorphonuclear neutrophils. These effects involve the action of several Yops including YopE, YopH, YopO/YpkA and YopT [4], [5]. One of the Yops, YopJ (named YopP in *Y. enterocolitica*) was shown to induce apoptotic cell death in *Yersinia*-infected macrophages *in vitro* [6]–[10] as well as in animal models of infection [11]–[13]. Induction of the apoptotic process was found to occur through the inhibition of NF-κB activation [14], [15] and MAPK signaling pathways [16]–[18], along with the activation of caspase pathways [10], [11], [19], [20]. These effects influence both inflammatory capacity and apoptosis of host immune cells.

It has been suggested that YopJ belongs to a family of proteases related to the ubiquitin-like protein proteases [21]. In line with this proposition, YopJ was shown to be a deubiquitinating cysteine protease capable of removing ubiquitin moieties from IκBα, thereby inhibiting its proteasomal degradation and leading to the down-regulation of NF-κB functions [22]. In addition, YopJ was recently demonstrated to acetylate Ser/Thr residues in the activation loop of MAPK kinases (MKKs) and IκB kinases (IKKs), consequently preventing their activation by phosphorylation [23], [24]. This newly identified acetyltransferase activity of YopJ may well account for its ability to inhibit MAPK pathways and NF-κB activation. It is noteworthy that current knowledge on the interactions between *Yersinia* and innate immune cells is mostly based on studies with *Yersinia* enteropathogenic species. Yet, in spite of the high homology between the effectors and the translocation apparatuses of these species and those of *Y. pestis*, it appears that certain mechanisms for evading host innate immunity differ between them. We have demonstrated that *Y. pestis* has a limited ability to induce programmed cell death in infected macrophages compared to *Y. enterocolitica* 0∶8 serotype [10], [25]. This observation was found to correlate with downgraded translocation of YopJ from *Y. pestis* to the target cell [10]. Similarly, whereas interactions of *Y. enterocolitica* 0∶8 with dendritic cells (DCs) lead to YopP-mediated induction of apoptotic cell death, infection of DCs with *Y. pestis* failed to affect cell viability [26], [27]. The difference in secretion levels of *Y. pseudotuberculosis* YopJ and *Y. enterocolitica* 0∶8 YopP was recently attributed to N-terminal sequence polymorphism between the proteins [13], and could also explain the limited secretion of *Y. pestis* YopJ.

The role of YopJ/YopP effectors in the *in vivo* virulence of enteropathogenic *Yersinia* is still uncertain. Several studies have reported impaired virulence of *Y. pseudotuberculosis yopJ* mutants [12], [13] and a *Y. enterocolitica yopP* mutant [28] in mouse models. However, others have reported that *yopJ* deletion had no effect on *Y. pseudotuberculosis* virulence [29]. The role of *Y. pestis* YopJ in pathogenesis was examined in several mouse models in which it was found to be dispensable for virulence. The LD_50_ of a *Y. pestis pgm^−^ yopJ* mutant following intravenous (i.v.) infection was only slightly higher (1.5-fold) than that of the parental strain [30]. In addition, a recent study in a rat model of bubonic plague has shown that YopJ was not essential for the manifestation of virulence [11]. Similar results were obtained in a mouse model of bubonic plague using a *yopJ* deletion mutant of the Kimberley53 strain [25].

Accumulating evidence seems to indicate that the ability to destroy host immune cells is not essential for the manifestation of virulence in *Y. pestis*. Moreover, the presence of viable infected immune cells at the infection site may rather allow the invading pathogen to replicate inside a shielded niche provided by the host cells. Given the fact that *Y. pestis* is found intracellularly during the early stages of infection [31], [32] and can replicate within macrophages [32], [33], one may assume that effective apoptotic activity against immune cells might impair its survival *in vivo*. The observation that *Y. pestis* demonstrates restricted capacity to induce apoptosis in macrophages and DCs, along with the finding that YopJ is not essential for *Y. pestis* virulence, led us to suggest that reduced apoptotic potency may have contributed to the highly pathogenic phenotype of *Y. pestis* [10].

In the present study, we challenged this assumption using the highly virulent *Y. pestis* Kimberley53, its *yopJ* deletion mutant and progeny derivatives expressing in trans either the *Y. pestis* YopJ or the YopP of *Y. enterocolitica* 0∶8. We demonstrate that while the YopP-expressing strain acquires potent apoptotic/cytotoxic capability against host macrophages, it appears to be less virulent than the parental or wild-type strains in the mouse model of bubonic plague (subcutaneous infection). However, the *Y. pestis*-YopP engineered strain is indistinguishable in its virulence in the septicemic or pneumonic mouse infection models (intravenous or intranasal administration, respectively). The inverse relationship between the cytotoxicity of YopP-expressing *Y. pestis* and its virulence following subcutaneous infection appears to also be associated with its ability to induce very rapid, effective and long-lasting immunoprotection against bubonic and pneumonic plague.

## Results

### *Y. pestis* overexpressing YopP is avirulent following subcutaneous administration to mice

**Figure 1:**
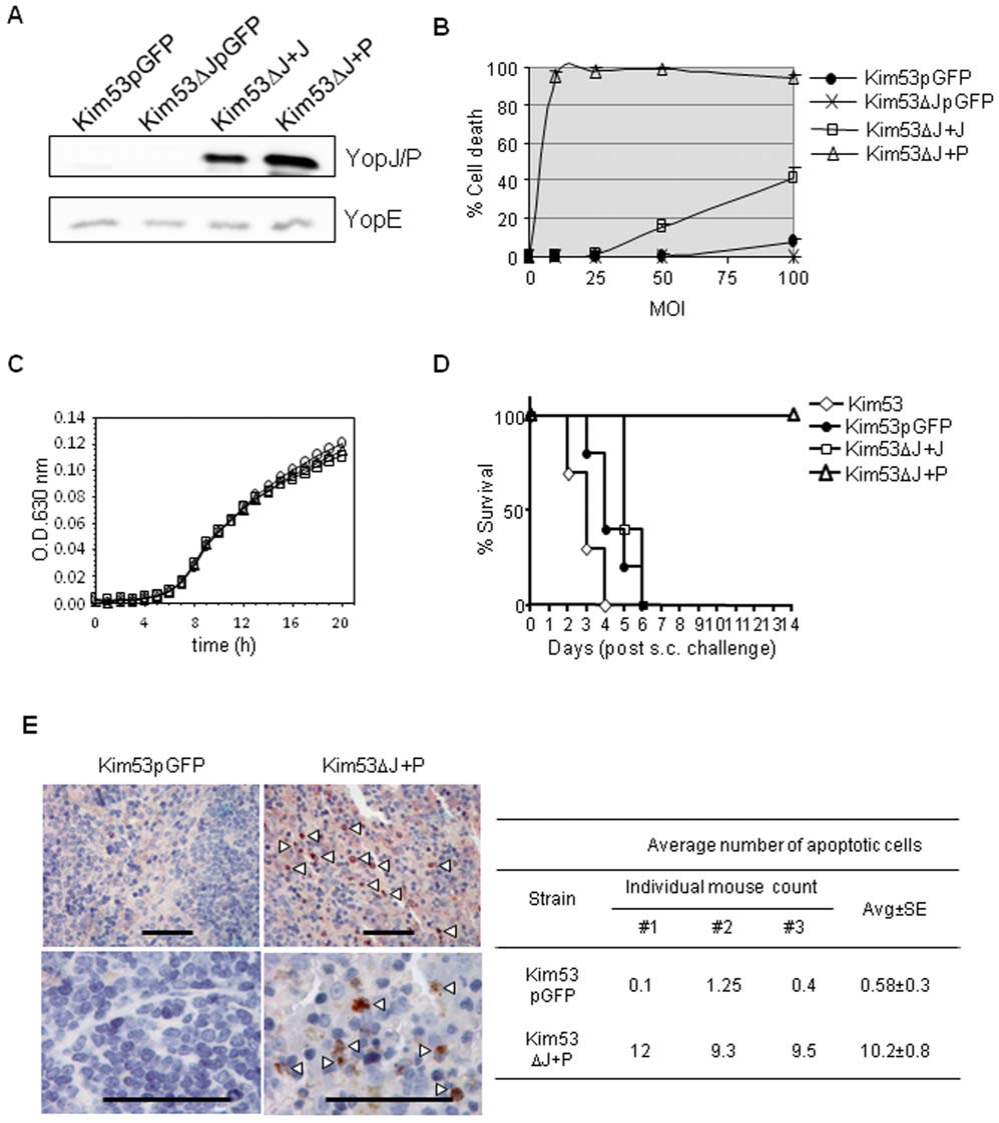
Expression of YopP in *Y. pestis* enhances its cytotoxicity in macrophages and reduces its virulence. (A) Assessment of bacterial YopJ/P expression. *Y. pestis* recombinant strains were grown for 3 hrs at 37°C. Western blot analysis was conducted with anti-YopJ/P antibodies (upper lane) or anti-YopE antibodies (lower lane). Endogenous YopJ expression in both wild-type Kim53 and Kim53pGFP strains could be detected only under non-standard conditions of considerably longer exposure time (data not shown). (B) Cytotoxic effect of *Y. pestis* recombinant strains on J774A.1 macrophage cell line. Cells were infected with Kim53pGFP (circle), Kim53ΔJpGFP (X symbol), Kim53ΔJ+J (square) and Kim53ΔJ+P (triangle) at the indicated MOIs for 1 hour. Cell death was determined by LDH release test. (C) Growth curves of Kim53pGFP (circle), Kim53ΔJ+J (square) and Kim53ΔJ+P (triangle). Bacteria were inoculated to OD_630_ 0.005 in heart infusion broth-containing microplate wells and incubated in a plate reader (Sunrise, Tecan) for an additional 22 hrs at 28°C. (D) Attenuation of YopP-expressing *Y. pestis* recombinant strain. Groups of mice were infected subcutaneously with 1×10^2^ cfu of Kim53 (10 mice per group, white diamond), Kim53pGFP (5 mice per group, black circle) or Kim53ΔJ+J (5 mice per group, white square), or with 1×10^6^ cfu of Kim53ΔJ+P (5 mice per group, white triangle). Mortality rates were followed daily for 14 days after infection. (E) Spleen sections, isolated from mice 48 hrs after s.c. infection with 1×10^4^ cfu of Kim53pGFP or Kim53ΔJ+P, stained with anti-active caspase-3 antibodies (left panel). Scale bar = 50 µm. The arrow head indicates apoptotic cells. Caspase-positive cells in at least 20 random non-overlapping microscopic fields (magnification, ×1000) were counted (three mice per group, right panel).

Expression of YopP, the YopJ homologue in *Y. enterocolitica* O∶8, in the background of the attenuated *Y. pestis* EV76 strain was previously shown to render this strain highly cytotoxic to J774A.1 and RAW 264.7 macrophage cells [10]. In order to explore whether enhanced cytotoxic potency would attenuate *Y. pestis* virulence, in this study, we used the highly virulent *yopJ*-deleted strain of *Y. pestis* Kimberley53 (Kim53ΔJ) [25] to generate recombinant strains expressing in trans either YopP (Kim53ΔJ+P) or YopJ (Kim53ΔJ+J) (Table 1). Plasmid construction was based on replacing the green fluorescent protein (GFP) gene from the pUC19 derivative plasmid pGFPuv by *yopP* or *yopJ* genes, under the control of the *lac* promoter. As a control, we used wild-type Kim53 carrying the same expression vector with the *gfp* gene (Kim53pGFP). As was shown in the past with the attenuated EV76-based strains [10], the newly constructed Kim53-derived strains exhibited expression levels comparable to those of the two homologous effectors (YopJ and YopP), but with pronounced differences in induction of cell death upon infection of macrophages. Expression of YopP conferred high cytotoxic potential, which expression of YopJ did not (Figure 1A, upper lanes and 1B). It should be noted that expression of YopJ/P had no apparent effect on the bacterial growth rate *in vitro* (Figure 1C).

**Table 1:** Bacterial strains and plasmids used in this study.

Strains and plasmids	Relevant characteristics	Reference or source
***Y. pestis*** ** strains**		
Kim53	Virulent, Kimberley53	[55]
Kim53ΔJ	Kim53 deleted for *yopJ*	[25]
Kim53pGFP	Kim53 carrying pGFPuv	This study
Kim53ΔJ+P	Kim53Δ*yopJ* carrying pYopP	This study
Kim53ΔJ+J	Kim53Δ*yopJ* carrying pYopJ	This study
Kim53Δp70Δp10	Spontaneously pPCP1- and pCD1-cured Kim53	[51]
EV76	*pgm^−^* (Girard's strain)	[56]
EV76ΔJ+P	EV76Δ*yopJ* carrying pYopP	[10]
**Plasmids**		
pGFPuv	*Lac-*controlled *gfp* gene on pUC vector; Amp*^R^*	CLONTECH, USA
pYopP	pGFPuv in which *gfp* gene was replaced by *Y. enterocolitica* O∶8 *yopP* gene	[10]
pYopJ	pGFPuv in which *gfp* gene was replaced by *Y. pestis* Kimberley53 *yopJ* gene	[10]

The impact of increased cytotoxicity on *Y. pestis* virulence was tested initially by subcutaneous (s.c.) infection of mice. Mice infected with 100 colony forming units (cfu) of Kim53ΔJ+P (equivalent to 100 LD_50_ of wild type Kim53) exhibited 100% survival, as opposed to 100% mortality in mice infected with an identical dose of the control Kim53pGFP or the wild-type Kim53 strain (Figure 1D). The effect of YopP expression was so dramatic that even after infection with 1×10^6^ cfu of Kim53ΔJ+P, all animals survived (Figure 1D). These results were observed with two independent isolates of Kim53ΔJ+P. Increasing the infection dose of Kim53ΔJ+P to 10^7^ cfu resulted in 75% survival, indicating that the Kim53ΔJ+P LD_50_ value is above 10^7^ cfu. At the same time, the less cytotoxic strain over-expressing YopJ (Kim53ΔJ+J; Figure 1B) remained virulent, as reflected by the mortality of infected mice (Figure 1D) and the calculated LD_50_ value of 1 cfu. It should be noted that Kim53pGFP had similar LD_50_ as Kim53, however, the former had some delay in mean time to death (from 3 to 4.4 days, *P*<0.01).

All of the tested Kim53-derived *Y. pestis* strains carry the pMT1, pCD1, pPCP1 plasmids and the *pgm* locus, and preserved the functionality of their TTSS, as manifested by their calcium-dependent growth at 37°C. In addition, YopP expression had no apparent effect on the expression of other *Y. pestis* Yop effectors such as YopE (Figure 1A, lower lanes). Consistent with our *in vitro* observations, the YopP-expressing *Y. pestis* strain induced significantly higher levels of apoptosis in infected spleen cells compared to the virulent Kim53pGFP strain, as determined by staining with anti-active caspase-3 antibodies (Figure 1E). It therefore appears that concomitant with YopP-mediated enhanced cytotoxicity, the *Y. pestis* bacteria expressing YopP exhibit reduced virulence in the bubonic plague model.

### Overexpression of YopP by *Y. pestis* affects bacterial colonization of internal organs

**Figure 2:**
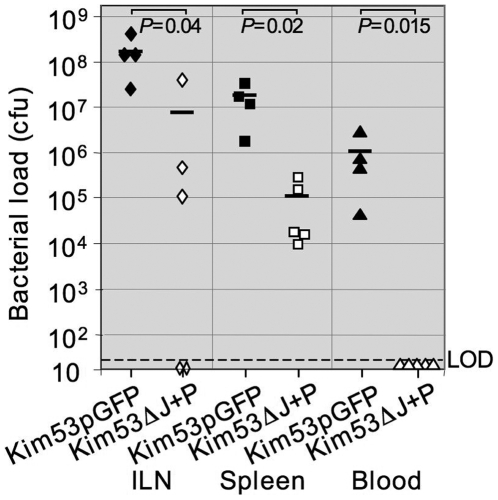
Kim53ΔJ+P is deficient in dissemination to target organs and to the blood following subcutaneous infection. Mice were infected subcutaneously with 1×10^4^ cfu of either Kim53pGFP (black symbols) or Kim53ΔJ+P (white symbols). Animals (4–5 per group) were sacrificed at 60 hours post-infection. Blood was collected, and the draining inguinal lymph nodes (ILN) and spleens were harvested, homogenized in 1 ml PBS and cultured on brain heart infusion agar plates at 28°C for 48 hrs. Values in Figure represent total bacterial loads in infected organs (cfu/organ), or bacterial concentration in blood (cfu/ml). LOD, limit of detection. Horizontal bars represent the average value of bacterial load in each case. Differences in bacterial concentrations in blood and internal organs were analyzed by the non-parametric Mann-Whitney test.

Host immune cells may serve *in vivo* as a favorable intracellular niche and a shielding vehicle for plague bacilli [31], [32], [33]. It was therefore interesting to determine whether the avirulent phenotype of Kim53ΔJ+P is associated with the cytotoxic effect of YopP through interference with bacterial propagation and delivery to internal tissues. Accordingly, we monitored the colonization of internal organs by Kim53ΔJ+P compared to the *Y. pestis* Kim53pGFP control strain. Mice were subcutaneously infected with 1×10^4^ cfu of each strain. Dissemination to draining inguinal lymph nodes (ILNs), the spleen and blood was examined 60 hours post-infection, a time point representing terminal stages of disease in Kim53pGFP-infected mice that served as controls. The number of colony forming units in the ILNs and the spleen of mice infected with Kim53ΔJ+P was about 100-fold lower than with the control *Y. pestis* strain (Figure 2). An even more pronounced disparity was observed in blood. Kim53ΔJ+P could not be detected in blood whereas the *Y. pestis* control strain reached an average concentration of 1×10^6^ cfu/ml. Interestingly, despite the fact that colonization of the spleen in Kim53ΔJ+P-infected mice reaches substantial bacterial count (1×10^5^ bacteria per spleen), the infection with high dose of Kim53ΔJ+P is not lethal (Figure 1D).

### Kim53ΔJ+P is highly virulent when administered through the intravenous or intranasal routes

**Figure 3:**
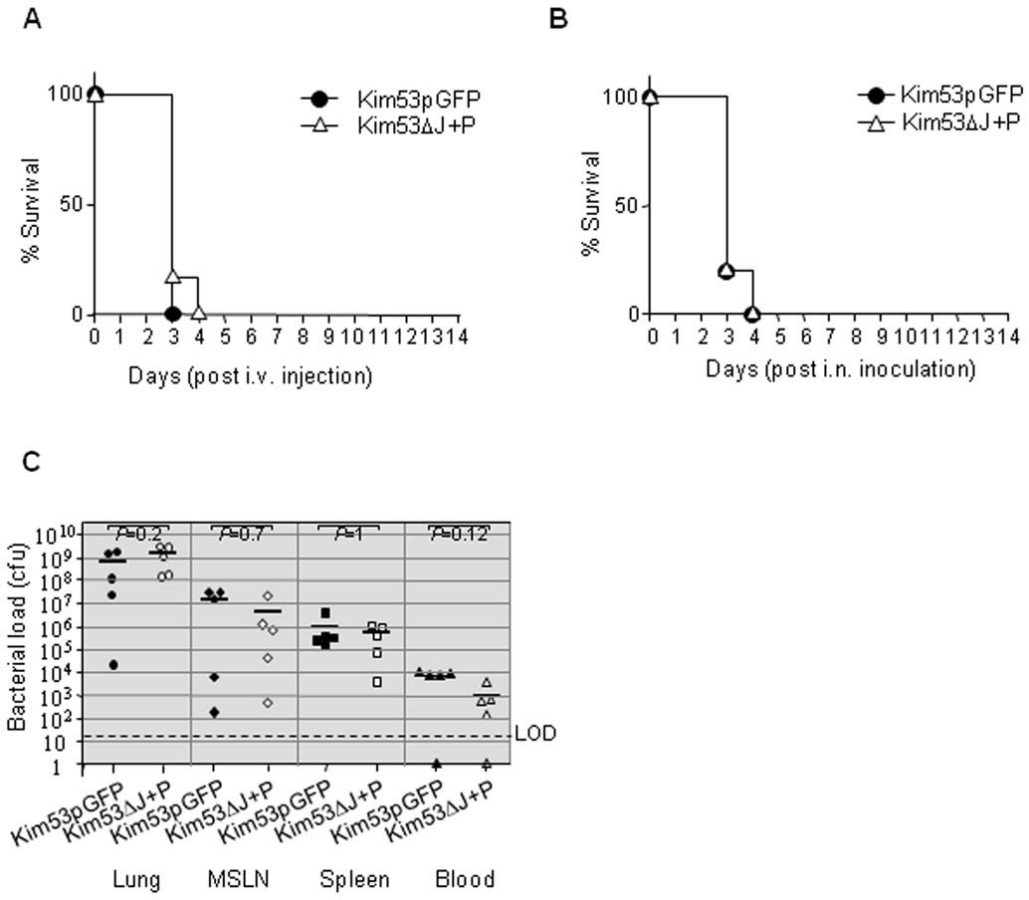
Kim53ΔJ+P is highly virulent following systemic or airway infection. (A) Systemic infection (i.v. inoculation). Groups of 6 mice were infected intravenously with a dose of 2×10^3^ cfu of Kim53pGFP (circle) or Kim53ΔJ+P (triangle), and mortality was monitored daily for 14 days. (B) Airway infection (i.n. inoculation). Groups of 5 mice were infected intranasally with 6×10^4^ cfu of Kim53pGFP (circle) or Kim53ΔJ+P (triangle). (C) Dissemination of *Y. pestis* strains to blood and internal organs following intranasal infection. Groups of 5 mice were infected intranasally with 2×10^5^ cfu of Kim53pGFP (black symbol) or Kim53ΔJ+P (white symbol) and sacrificed 48 hours post-infection. Bacterial concentration in blood and total bacterial loads in lungs, mediastinal lymph nodes (MSLNs) and the spleen were determined as described in the legend to Figure 2.

In an attempt to gain deeper insight into the mechanisms involved in the observed attenuation of virulence of the Kim53ΔJ+P strain, mice were infected with 2,000 cfu intravenously (i.v.), thereby bypassing the subcutaneous barrier. Under these conditions, both *Y. pestis* strains, the YopP-expressing strain and the Kim53pGFP strain, caused 100% mortality with comparable mean time to death (MTTD) of 3.2 and 3.4 days, respectively (Figure 3A).

In view of these results, it became interesting to examine whether Kim53ΔJ+P would also exhibit an attenuated phenotype under the conditions of respiratory infection. Since the role of *Y. pestis* YopJ in the development of primary pneumonic plague has not yet been examined, we first compared the virulence of Kim53ΔJ to that of wild-type Kim53 using intranasal (i.n.) infection of mice. Both strains demonstrated identical LD_50_ values (ca. 550 cfu), consistent with the results obtained with other virulent wild-type *Y. pestis* strains harboring an intact *yopJ* [34], [35]. This finding indicates that YopJ is not essential for virulence via the airways route.

In experiments aimed at assessing the respiratory virulence of Kim53ΔJ+P, it was found that all mice infected intranasally with 6×10^4^ cfu of Kim53ΔJ+P (equivalent to ∼30 LD_50_ of the wild-type strain), died within four days at the same rate as those infected intranasally with the virulent control strain Kim53pGFP (Figure 3B). In addition, both strains demonstrated the same i.n. LD_50_ value of 1,800 cfu, which is comparable to that of the parental Kim53 and Kim53ΔJ strains. Moreover, disease progression and systemic bacterial dissemination, as reflected by bacterial cfu counts in the lungs, mediastinal lymph nodes (MSLNs), spleen and blood, were also comparable (Figure 3C). It may be of interest to note in this context that macrophages from alveolar origin (MH-S) were found to be more resistant to the cytotoxic effect induced by Kim53ΔJ+P (reduction of 80% in cell death upon infection with 10 MOI) compared to J774A.1 macrophage cells.

### The non-virulent phenotype of Kim53ΔJ+P is dominant over the virulent phenotype of Kim53pGFP in subcutaneous co-infections

One possible explanation for the reduced infectivity and spread of Kim53ΔJ+P following s.c infection is that this strain kills the transporter immune cells and thus prevents dissemination. An alternative explanation is that these bacteria induce a rapid and efficient protective immune response that eliminates the bacteria from the bloodstream and accounts for the attenuated virulence phenotype.

**Figure 4:**
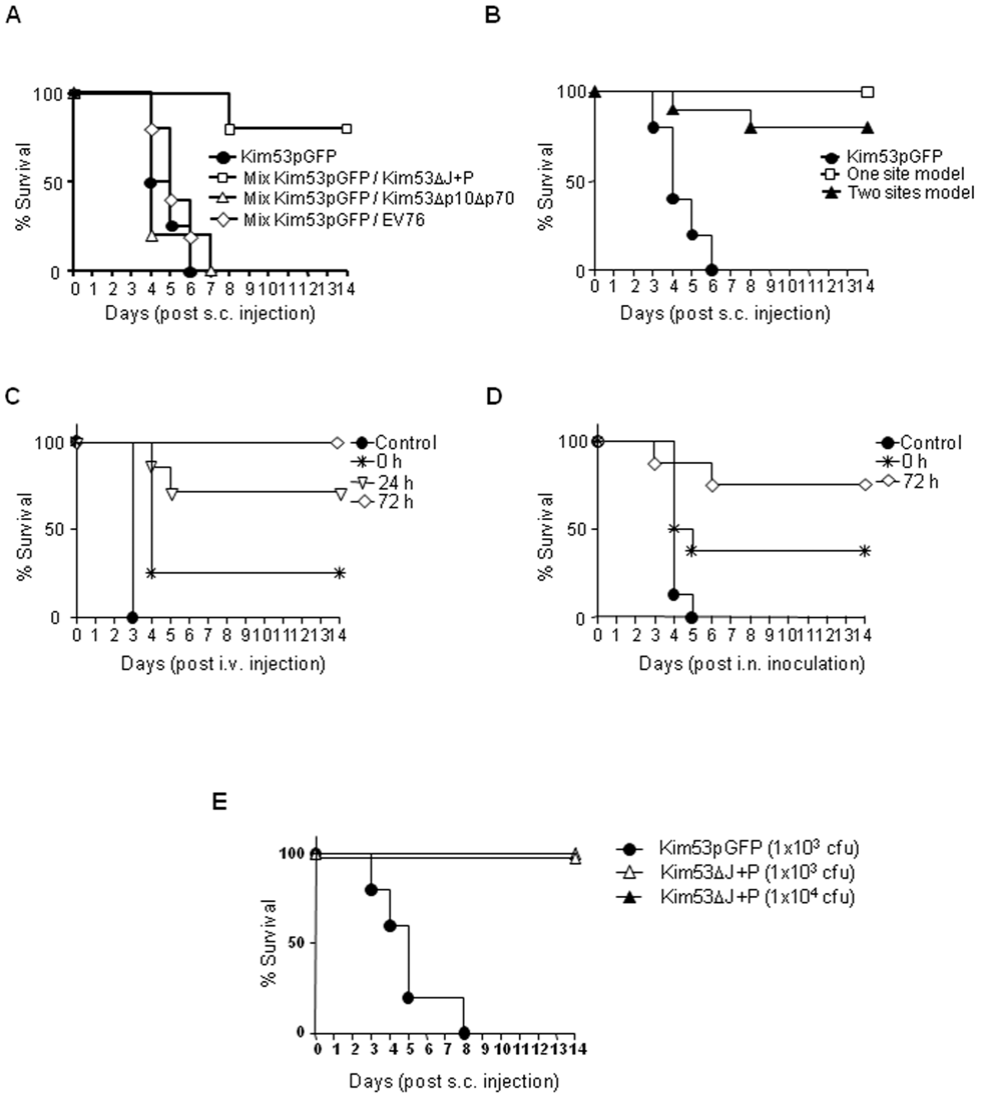
Subcutaneous administration of Kim53ΔJ+P protects mice coinfected with a virulent *Y. pestis* strain. (A) Subcutaneous co-infections of the virulent Kim53pGFP and various *Y. pestis* strains were administered at a single site. Groups of 5 mice were infected at a single site located on the lower back with 1×10^2^ cfu of Kim53pGFP alone (circle); a mixture of 1×10^2^ cfu of Kim53pGFP with 1×10^3^ cfu of Kim53ΔJ+P (square); 1×10^3^ cfu of Kim53Δp10Δp70 (triangle); or 1×10^3^ cfu of *Y. pestis pgm^−^* EV76 strain (diamond). Mortality was monitored daily for 14 days (B). Comparison of subcutaneous coinfection with Kim53ΔJ+P and Kim53pGFP administered at a single site (square) and at two separate sites (black triangle). Mice were injected in their lower back with a mixture of Kim53pGFP (1×10^2^ cfu) and Kim53ΔJ+P (3×10^3^ cfu) for the single site. For the two separate sites, 10 mice were injected in the upper back with 3×10^3^ cfu of Kim53ΔJ+P and immediately thereafter with 1×10^2^ cfu of Kim53pGFP in the lower back. Control mice were injected in the lower back with 1×10^2^ cfu of Kim53pGFP (circle). (C) Subcutaneous infection of mice with Kim53ΔJ+P followed by intravenous challenge with virulent *Y. pestis*. In total, 8 mice were injected subcutaneously in the lower back with 10^4^ cfu of Kim53ΔJ+P. Afterwards, 2×10^3^ cfu of the virulent Kim53pGFP were administered intravenously immediately (0 hr), or after 24 hrs or 72 hrs. Control mice were only infected intravenously with Kim53pGFP (circle). (D) Subcutaneous infection of mice with Kim53ΔJ+P followed by intranasal challenge with virulent *Y. pestis*. In total, 8 mice were injected subcutaneously with Kim53ΔJ+P as described in (C) and exposed intranasally to 6×10^3^ cfu (∼3LD_50_) of the virulent Kim53pGFP immediately (0 hr) or 72 hrs later. Mortality was monitored daily for 14 days. (E) Subcutaneous infections of K^b^D^b−/−^ mice (class I MHC genes deleted) with 1×10^3^ cfu of Kim53pGFP (circle), 1×10^3^ cfu of Kim53ΔJ+P (open triangle) or 1×10^4^ cfu of Kim53ΔJ+P (closed triangle).

To clarify this issue, we co-infected mice subcutaneously with Kim53ΔJ+P and the virulent Kim53pGFP strain (Table 2). Indeed, co-infection with Kim53ΔJ+P could completely protect animals from 10, 100 and even 10,000 LD_50_ of the virulent Kim53pGFP strain. Such protection could be achieved as long as the ratio of Kim53ΔJ+P to Kim53pGFP was at least 10∶1. Lowering the ratio between the two strains to 1∶1 decreased the extent of protection (Table 2). Such protection against subcutaneous infection with Kim53pGFP could not be provided by co-infection with different attenuated *Y. pestis* strains such as Kim53Δp10Δp70 and EV76 (Table 1, Fig. 4A). To examine whether the Kim53ΔJ+P-mediated resistance to lethal infection with Kim53 is a systemic or a localized effect, the animals were simultaneously infected at separate sites, with each site inoculated subcutaneously with a different strain. As shown in Figure 4B, even under conditions of distant s.c inoculation sites, the ability of Kim53ΔJ+P to counteract the lethality of Kim53pGFP was substantial. It therefore appears that s.c infection with Kim53ΔJ+P leads to the development of rapid systemic resistance against a subcutaneous challenge that would otherwise kill the animal within 6 days.

**Table 2:** Protection conferred by Kim53ΔJ+P upon co-infection of mice with virulent Kim53pGFP.

Bacterial ratio	Bacterial dose (cfu)		% Survival (Live/Total)^a^
	Kim53pGFP^b^	Kim53ΔJ+P	
1∶100	10	10^3^	100 (5/5)
	10^2^	10^3^	80 (4/5)
1∶10	10^3^	10^4^	80 (4/5)
	10^4^	10^5^	80 (4/5)
1∶1	10^2^	10^2^	20 (1/5)
	10^3^	10^3^	20 (1/5)
-	10	-	0 (0/5)

a Mice were co-infected subcutaneously with mixtures of Kim53pGFP and Kim53ΔJ+P in different bacterial ratios. Mortality was monitored for 14 days.

b One colony forming unit is equivalent to 1 s.c. LD_50_.

### Subcutaneous administration of Kim53ΔJ+P produces rapid and effective systemic resistance against lethal intravenous and intranasal challenges with a virulent *Y. pestis* strain

We assessed the time window for the induction, duration and extent of the systemic resistance generated by s.c administration of Kim53ΔJ+P. The first series of experiments was based on subcutaneous infection of mice with 1×10^4^ cfu of Kim53ΔJ+P, followed by i.v. challenge at different times with ∼65 LD_50_ of Kim53pGFP (the septicemic model). Whereas all mice in the control group (pretreated with saline) died within 3 days following i.v. challenge, mice pretreated with s.c. inoculation of Kim53ΔJ+P were protected in a time-dependent manner. Thus, 25%, 71% and 100% protection levels were observed at time intervals of 0, 24 and 72 hours, respectively (Figure 4C). It is noteworthy that even simultaneous co-infection of Kim53ΔJ+P and the virulent Kim53pGFP already provided a certain degree of protection, and that pretreatment with Kim53ΔJ+P induced rapid and complete protection within 3 days after inoculation.

Encouraged by these results, we then studied the scenario of protection against airway infection (i.n. challenge), which serves as a model of pneumonic plague. All control mice died within 5 days after an i.n challenge with 6,000 cfu (∼3LD_50_) of Kim53pGFP. Relatively high protection levels were achieved in mice that were challenged simultaneously (38% survival) or 72 hours (75% survival) after s.c Kim53ΔJ+P pretreatment (Figure 4D; note that in the intranasal experiments the ratio of Kim53ΔJ+P to Kim53pGFP was only 2∶1). Three days after s.c. inoculation of the Kim53ΔJ+P cytotoxic strain, we could not detect any development of antibodies against the highly immunogenic F1 antigen of *Y. pestis*.

**Figure 5:**
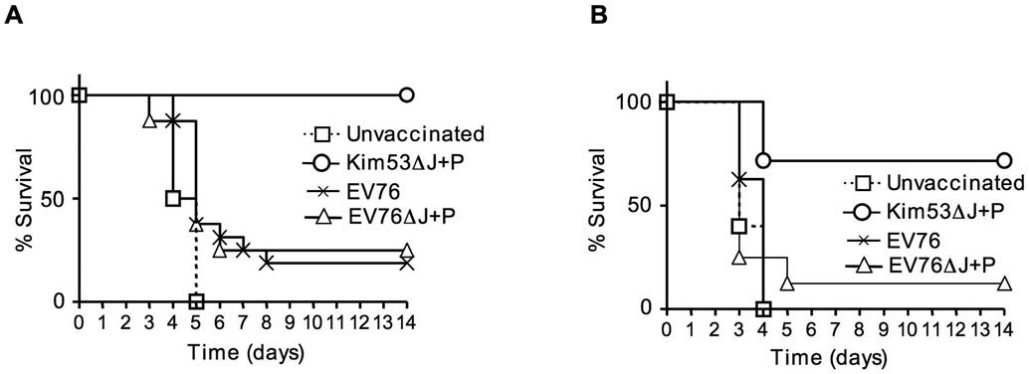
*Y. pestis* Kim53ΔJ+P strain confers prolonged protection against plague. (A) Mice (7–8 per group) were infected subcutaneously with 1×10^5^ cfu of Kim53ΔJ+P (circle); EV76 (× symbol); or EV76ΔJ+P (triangle). Control animals were treated with saline (square). Animals were bled for ELISA determination of anti-F1 antibody titers 45 days following immunization. Titers following immunization with Kim53ΔJ+P ranged from 64,000 to 100,000, with GMT of 18,500, while titers for the EV76 or EV76ΔJ+P immunized animals ranged from 640 to less than 10, with GMT of 30 and 10, respectively. Animals were challenged subcutaneously with 1×10^3^ cfu (∼1×10^3^ LD_50_) of *Y. pestis* Kim53 (panel A) or intranasally with 8×10^3^ cfu (∼15 LD_50_) of *Y. pestis* Kim53 (panel B).

Certain characteristics of Kim53ΔJ+P-induced resistance are reminiscent of the cross-priming mechanism of CD8 T cell activation [36]–[38]. To examine the involvement of CD8 T cells in the protective mechanism induced by Kim53ΔJ+P, C57BL/6J mice and their isogenic strain deleted for class I MHC genes (K^b^D^b−/−^) were infected subcutaneously with the virulent Kim53pGFP or the cytotoxic Kim53ΔJ+P strain (Figure 5E). All mice infected with Kim53ΔJ+P survived the infection, whereas all mice infected with Kim53pGFP died. This finding indicated that the observed attenuation of the cytotoxic strain is not a result of a cross-priming mechanism and CD8 T cell activation. In addition, inflammatory processes in the spleen did not seem to account for the rapid development of systemic protection, since no significant differences could be demonstrated between the levels of pro-inflammatory cytokines (TNF-α and IL-6) and Th1 cytokines (IFN-γ, IL-2 and IL-12) at 36, 60 and 72 hours post infection with Kim53ΔJ+P or Kim53pGFP (data not shown).

### Rapid induction of resistance by subcutaneous administration of Kim53ΔJ+P does not interfere with the development of long-term adaptive immunity

The possible consequences of the rapid induction of resistance by Kim53ΔJ+P on the development of long-term humoral immunity were studied. In these experiments, the Kim53ΔJ+P strain simulated a vaccinating agent while the wild type *Y. pestis* Kim53 served as a challenging agent. ELISA titers of anti-F1 antibodies served as a marker for the development of protective long-lasting immunity. Results were compared to those obtained in mice that had been vaccinated with the EV76 vaccine strain. In the mouse model of bubonic plague, the assay conditions were based on a single subcutaneous dose (1×10^5^ cfu) of Kim53ΔJ+P or EV76, followed by subcutaneous challenge with 1×10^3^ LD_50_ of *Y. pestis* Kim53 45 days later. Mice injected with saline served as the control group. Under these experimental conditions, the EV76 strain failed to elicit significant protective immunity, whereas all mice vaccinated with Kim53ΔJ+P survived (Figure 5A). In the mouse model of pneumonic plague, subcutaneous vaccination with the Kim53ΔJ+P strain was followed 45 days later by i.n. challenge with virulent *Y. pestis* Kim53. Under challenge with 5.5×10^3^ cfu, which is equivalent to about 15 LD_50_ (Figure 5B), 75% of the Kim53ΔJ+P-vaccinated mice survived, whereas all control mice and mice vaccinated with the EV76 strain died.

ELISA titers of anti-F1 antibodies at 45 days after subcutaneous infection with 1×10^5^ cfu of Kim53ΔJ+P ranged from 6,400 to 100,000, with a geometric mean of 23,000. Under the same conditions of inoculation with EV76, the anti-F1 titers ranged from below the limit of detection (<10) to 320, with a geometric mean of less than 30. Hence, rapid induction of resistance by subcutaneous administration of Kim53ΔJ+P does not seem to interfere with the induction of long-term adaptive immunity against the plague. Rather, this route of exposure to Kim53ΔJ+P appears to be a means for improved stimulation of long-term adaptive immunity.

## Discussion

The ability of bacterial pathogens to induce apoptosis in target immune cells such as macrophages and neutrophils provides an obvious advantage during infection, since these cells would otherwise kill the pathogens. Nevertheless, under particular circumstances, preventing apoptosis can provide a survival advantage by allowing the bacteria to replicate inside a shielded niche and eventually to be transported to target organs while avoiding recognition by the immune system. Under such circumstances, an effective mechanism of cell killing at the early stages of infection would therefore be disadvantageous. *Y. pestis* has long been considered to be a facultative intracellular pathogen [1], [39] and several studies have demonstrated that the pathogen survives and replicates in macrophages both *in vivo* and *in vitro* [31], [33], [40]–[45]. It is believed that *Y. pestis* replicates within macrophages during the early stages of infection at peripheral host sites, while extracellular growth is predominant during the later stages of infection.

*Y. pestis* exploits a variety of mechanisms for evading or coping with the host immune responses in the early stages of infection [1], [5], [39]. Currently known mechanisms include lipid A alternation to prevent the stimulation of LPS-induced inflammatory response [46] and virulence mechanisms involving TTSS effectors and other factors such as Pla [47].

The TTSS effector YopJ/P of *Yersinia* was shown to exert a variety of adverse effects upon interaction with innate immune cells, including induction of apoptotic cell death [6]–[8]. However, our recent *in vitro* studies with the *Y. pestis* EV76 strain demonstrated ineffective YopJ-mediated cytotoxicity of *Y. pestis* in macrophages and dendritic cells, due to inefficient translocation of the effector to target cells [10], [26]. In contrast, YopP (the homologue of YopJ) from *Y. enterocolitica* O∶8 was shown to be readily translocated into target cells and to exert high cytotoxic activity. These features were also preserved when YopP was expressed in a *Y. pestis* genetic background [10]. The essentiality of YopJ/P functions for *Yersinia* virulence *in vivo* is not yet clear. Studies on the involvement of YopJ in *Y. pestis* virulence indicated that this effector was not essential for virulence in rodent models of septicemic and bubonic plague [10], [11], [30]. Here we show that YopJ is also not essential for virulence in the mouse model of pneumonic plague, as reflected by the finding that a *yopJ*-deleted Kim53 mutant fully retains its virulence upon intranasal administration. We therefore assumed that the impaired cytotoxicity of *Y. pestis* may rather contribute to virulence by restraining YopJ translocation into phagocytic cells and thereby limiting the ability to induce cell death, although the ability to suppress MAPK phosphorylation and TNF-α secretion is retained [10].

To examine this hypothetical relationship between cytotoxicity and virulence, we generated *Y. pestis* strains expressing YopP or YopJ (Kim53ΔJ+P, Kim53ΔJ+J) and assessed their cytotoxic activity and virulence in mouse models of plague disease, comparing them to the control virulent *Y. pestis* strain (Kim53pGFP). We show that *Y. pestis* carrying the expression vector alone, the *yopJ* deletion mutant and the YopJ-expressing strain (Kim53ΔJ+J) all exhibit low cytotoxic potency, and preserve their virulence upon subcutaneous infection of mice (Figure 1D). In contrast, the Kim53ΔJ+P strain appeared to acquire markedly enhanced cytotoxic activity in its interaction with J774A.1 macrophage cells *in vitro* and in the tissue of infected mice (Figure 1B, E and 2). Concomitant with this enhanced cytotoxicity, the subcutaneously administered YopP-expressing *Y. pestis* strain exhibited a dramatic decrease in virulence, as reflected by elevation of the LD_50_ value from 1 cfu to more than 10^7^ cfu. The colonization of internal organs by the YopP-expressing *Y. pestis* strain was substantially reduced (∼100-fold decrease relative to the control strain) and most significantly, bacteria could not be detected at all in the blood (Figure 2). The mere presence of a high load of plasmid copies or high levels of Yop effector (mediated by their expression from the expression vector) could not account for the observed attenuated phenotype, since the control strain (Kim53pGFP) carrying the same vector load and the YopJ-expressing strain (Kim53ΔJ+J) were highly virulent similar to the wild-type Kim53 strain. In addition, extensive control experiments indicated that expression of YopP did not affect the functionality of TTSS, the bacterial growth rate or pYopP plasmid stability (Figure 1A, C and Materials and Methods). It appears therefore that an inverse relationship between YopP-induced cytotoxicity and virulence upon infection via the subcutaneous route does exist. A similar trend was recently reported for a recombinant *Y. pseudotuberculosis* strain expressing *Y. enterocolitica* YopP, which demonstrated impaired virulence in oral mouse infections [13]. Other examples of an inverse relationship between induction of macrophage apoptosis and virulence were also demonstrated in other pathogens such as *Francisella tularensis* [48] and *Legionella pneumophila* [49].

It is quite striking however that the virulence of Kim53ΔJ+P was preserved when it was inoculated via the intravenous and intranasal routes of infection (Figure 3). These results may suggest that the Kim53ΔJ+P strain is not genetically impaired either in its virulence potential or in its ability to disseminate to various organs. Rather, they emphasize the uniqueness of the subcutaneous mode of infection for the manifestation of the *Y. pestis* attenuated phenotype.

One possible explanation for this site-specific phenotypic attenuation is that the subcutaneous administration of Kim53ΔJ+P may induce a rapid immune protective response. This notion is indeed supported by the studies of mixed subcutaneous infections of a virulent *Y. pestis* strain and Kim53ΔJ+P. The cross-protection provided by Kim53ΔJ+P was so effective that it could overcome infection with at least 10^4^ LD_50_ of the virulent strain (depending on the cfu ratio of Kim53ΔJ+P to virulent Kim53pGFP; Figure 4A and Table 2). Two possible mechanisms could account for this cross-protection: a localized destruction of co-infected immune cells leading to disruption of the propagation of *Y. pestis*, or alternatively induction of certain resistance mechanisms. The fact that effective cross-protection could be mediated by subcutaneous inoculation of Kim53ΔJ+P even when the co-infecting virulent strain was administrated at a separate site supports the idea of systemic protection (Figure 4B). Most striking was the observation that effective cross-protection against intravenous and intranasal lethal challenges of the virulent strain was acquired by subcutaneous administration of Kim53ΔJ+P (Figure 4C, D). Furthermore, since the mean time to death of naïve mice infected intravenously or intranasally with virulent *Y. pestis* is 3–4 days, it appears that the protective response developed very rapidly. This was clearly demonstrated when the cytotoxic Kim53ΔJ+P strain was administered 72 hours prior to the challenge with the virulent Kim53pGFP strain.

The association between the enhanced apoptotic effect of Kim53ΔJ+P and the rapidly acquired protection is reminiscent of the proposed MHC-I-mediated cross-priming mechanism of CD8 T cell activation, which is initiated by pathogen-induced apoptosis [36], [50]. In this process, bacterial engulfment by macrophages leads to the formation of apoptotic vesicles that contain bacterial antigens. These vesicles are then engulfed and processed by neighboring dendritic cells, leading downstream to the rapid activation of CD8 T cells. However, our results show that the cytotoxic Kim53ΔJ+P strain retained the attenuated phenotype upon subcutaneous infection of mice deleted for class I MHC genes (K^b^D^b−/−^), indicating that CD8 T cell activation cannot account for the dramatic attenuation of virulence in the Kim53ΔJ+P strain. The mechanistic basis underlying the enhancement of the rapid protective response by subcutaneous administration of Kim53ΔJ+P therefore remains to be determined, and is probably related to a combination of innate and adaptive immunity.

Our data indicate that s.c. administration of Kim53ΔJ+P can elicit a robust long-term immune response. This was demonstrated by (a) development of 1,000-fold higher anti-F1 antibody titers compared to those obtained by immunization with a similar dose of the EV76 vaccine, and (b) ability to achieve considerably more effective and long-lasting protective immunity compared to the EV76 vaccine strain, in both the bubonic and pneumonic plague mouse models (Fig. 5). Nevertheless, in spite of the observation that EV76ΔJ+P is more cytotoxic to macrophages than EV76, both strains are equally poor in their ability to elicit specific anti-F1 antibody response or to provide protection. These results suggest that expression of YopP per se and its ability to confer extensive cytotoxicity are not sufficient to explain the potent immune response demonstrated by Kim53ΔJ+P. Irrespective of the actual mechanisms of immunity, the observed development of rapid and long-lasting immunity and the importance of the inoculation site may have practical implications in the design of future *Y. pestis* vaccines/therapies against pneumonic plague.

The downgraded translocation efficiency of YopJ may represent an additional example of a virulence mechanism developed by *Y. pestis*. Retention of YopJ expression in *Y. pestis* indicates that the low amounts of protein translocated into target cells may still provide a survival advantage to *Y. pestis* during *in vivo* infection [10], [11]. It is therefore tempting to speculate that the diminished cytotoxicity of YopJ was evolved to fit the unique life cycle of *Y. pestis* in nature. Thus, following the intra-dermal injection of *Y. pestis* bacteria by fleas, the ineffective YopJ-mediated cytotoxicity ensures evasion from the rapid induction of immune response, which otherwise could prevent systemic infection (as is the case when *Y. pestis* expresses YopP rather than the endogenous YopJ). Obviously, the absence of bacteria in the blood will terminate the *Y. pestis* cycle in nature since the vector fleas must feed on a septicemic mammal.

## Materials and Methods

### Bacterial strains

*Y. pestis* strains used in this study are listed in Table 1. Plasmid construction was based on replacing the GFP gene in pGFPuv plasmid (Clontech) by *yopP* or *yopJ* genes of either *Y. enterocolitica* O∶8 or *Y. pestis*, respectively. Construction of the Kimberley53 *yopJ* deletion mutant (Kim53ΔJ) was performed as described previously [10]. The pGFPuv, pYopJ and pYopP plasmids were introduced into *Y. pestis* strains by electroporation resulting in the generation of Kim53pGFP, Kim53ΔJ+J and Kim53ΔJ+P strains, respectively. These strains were routinely grown in heart infusion broth (HIB, Difco) supplemented with 100 µg/ml ampicillin (Sigma, Israel). The in vivo stability of pGFPuv, pYopJ and pYopP was examined by plating bacteria recovered from the spleen and the draining lymph nodes up to 7 days post infection, on nonselective and selective plates (100 µg ampicillin, the antibiotic marker on pGFPuv and its derivatives). All bacteria recovered from mice infected with the Kim53pGFP, Kim53ΔJ+J or Kim53ΔJ+P strains were found to be resistant to ampicillin. The Kim53 strain carrying the pGFPuv plasmid (Kim53pGFP) demonstrated comparable LD_50_ values and a mean-time-to-death (MTTD) to the wild type strain via both s.c. and i.n. routes of infection and served as a control strain. Genotype verification of all obtained phenotypes was done by PCR and Western blot analyses using rabbit anti-YopJ/P polyclonal antibodies [10]. All the strains used in this study were tested and verified to carry the plasmids and virulence markers pMT, pCD1, pPCP1 and the *pgm* locus.

### Cell cultures

The murine macrophage-like cell line J774A.1 and the murine alveolar macrophage cell line MH-S were obtained from ATCC. The J774A.1 cells were grown at 37°C under 5% CO_2_ in DMEM (Beit Haemek, Israel) supplemented with 10% heat-inactivated fetal calf serum (FCS), 1 mM sodium pyruvate, 4 mM L-glutamine and 1% non essential amino acids. MH-S cells were grown at 37°C under 5% CO_2_ in RPMI 1640 medium (Beit Haemek, Israel) supplemented with 10% heat-inactivated FCS, 1 mM sodium pyruvate, 2 mM L-glutamine, 10 mM HEPES (pH 7.3), 0.003% bicarbonate, 0.05 mM 2-mercaptoethanol.

### Cell cytotoxicity assay

For infection of cultured cells, bacteria were grown by shaking for 18 hours at 28°C in HIB. The resulting cultures were diluted in HIB medium to OD_660_ 0.05 and allowed to grow for 3 hours at 37°C to induce Yops secretion [10]. Bacteria were harvested, washed once and re-suspended in DMEM supplemented with 10% FCS and added to the cells (2–6×10^4^ cells/well) at the indicated multiplicities of infection (MOIs). Bacteria were impacted onto the cells by centrifugation at 130 g for 5 min followed by incubation for additional one hour at 37°C, 5% CO_2_. Gentamicin was then added to the cultures to a final concentration of 50 µg/ml and the cultures were thereafter incubated for additional 6 hours until harvesting. Supernatants of infected macrophages were quantified colorimetrically for released lactate dehydrogenase (LDH) using the Cytotox 96 non-radioactive cytotoxicity assay (Promega) according to the manufacturer's instructions. The absorbance (A_490_) was determined using a microplate reader (Sunrise, Tecan). Cytotoxicity caused by bacteria was expressed as % cell death. Percentage of cytotoxicity was calculated using the formula: 100×[(experimental release–spontaneous release)/(maximum release–spontaneous release)]. The spontaneous release reflects the amount of LDH released from the cytoplasm of uninfected macrophages, whereas the maximum release is the amount of LDH released from detergent-lysed uninfected macrophages.

### Western blot analysis

Bacteria (5×10^7^) were lysed with Laemmli Sample buffer (Bio-Rad) and subjected to 10% SDS-PAGE. After transfer to nitrocellulose membranes, duplicate membranes were developed with polyclonal rabbit anti-peptide YopP/J antibodies, which react with both YopP and YopJ as described by Zauberman et al. [10] or with polyclonal goat anti-YopE antibodies (bL-20, Santa Cruz Biotechnology) followed by horseradish peroxidase (HRP) conjugated second antibody and then visualized by electrochemical luminescence (ECL).

### Antisera and serological tests

Mouse anti-F1 antibody titer determination was performed by ELISA as described previously [51]. Briefly, microtiter plates were coated with purified recombinant F1 antigen [52]. Tested sera were serially diluted by 2-fold dilutions in a final volume of 50 µl and were incubated in the wells for 1 hour at 37°C. Alkaline phosphatase–labeled rabbit anti-mouse IgG or goat anti-mouse IgM (1/2500 dilution, Sigma) was used as the 2^nd^ layer. Titers were defined as reciprocal values of the endpoint serum dilutions, which displayed OD_405_ values two fold higher than normal serum controls.

### Histology and immunohistochemistry

Formalin-fixed spleens were embedded in paraffin and 2 µm sections were prepared. Apoptotic cells were then detected by immunohistochemical staining using anti cleaved caspase-3 polyclonal antibodies (Cell signaling) and the Histostem SP kit (Zymed), according to the manufacturer's instructions.

### Animal studies

All animal experiments were performed in accordance with the Israeli law and were approved by the Ethics Committee for animal experiments at the Israel Institute for Biological Research. Female 5–6 weeks old of OF1 mice (IFFA CREDO S.A., France), C57BL/6J (Harlan, Israel) and K^b^D^b−/−^ (received from Prof. L. Eisenbach, Weizmann institute, Israel) [53] were used in this study. All in vivo experiments were carried out in OF1 mice, unless otherwise noted. For subcutaneous (s.c.) and intravenous (i.v.) infections of mice, *Y. pestis* bacteria were grown on brain heart infusion agar (BHIA, Difco) for 48 hours at 28°C, suspended in 5 ml saline solution (0.9% NaCl), and then diluted in saline solution to the required infection dose.

Bacteria were quantified by counting colony forming units after plating and incubating on BHIA plates. In s.c. infections, samples of 100 µl were administered into the mouse lower back. Under these conditions the LD_50_ of the Kimberley53 strain is 1–3 cfu [51]. In i.v. infections, samples of 50 µl were injected into the mouse tail vein. For intranasal (i.n.) infections, *Y. pestis* strains were grown on BHIA as above and then suspended in HIB medium supplemented with 0.2% (+)Xylose (Sigma) and 2.5 mM CaCl_2_ (Sigma) to OD_660_ 0.01 and incubated for additional 22 hrs at 28°C in a shaker (100 rpm). At the end of the incubation period the cultures were washed, diluted in saline solution to the required infection dose and quantified by cfu counting as described above. Prior to infection, mice were anaesthetized with a mixture of 0.5% Ketamine HCl and 0.1% Xylazine and then infected intranasally with 35 µl/mouse of bacterial suspension. The intranasal LD_50_ of the Kimberley53 strain under these conditions is ca. 550 cfu. LD_50_ values were calculated according to the method described by Reed and Muench [54]. Mann-Whitney test was used to analyze significant differences in time to death and was complemented by the Log-rank test as a means to compare animal survival curves.

For bacterial dissemination to internal organs and blood, groups of 4–5 mice were anaesthetized, tail vein blood was collected and spleens, draining inguinal lymph nodes, lungs and mediastinal lymph nodes were then harvested and tissue homogenates were prepared in 1 ml PBS/organ. Bacterial enumeration in tissue homogenates or in blood samples was done by plating serial dilutions in PBS on BHIA and calculating cfu/organ or cfu/1 ml blood. Determination of TNF-α, IL-6, IFNγ, IL-2 and IL-12 concentrations in organs homogenates was performed by enzyme-linked immunosorbent assays using the mouse Duo Set kit (R&D systems). Differences in cytokines and bacterial concentrations in blood and internal organs were analyzed by the non-parametric Mann-Whitney test.
